# Evaluation of Drug-Disease Interactions and Their Association with Unplanned Hospital Readmission Utilizing STOPP Version 2 Criteria

**DOI:** 10.3390/geriatrics2040033

**Published:** 2017-11-08

**Authors:** Mandy Hau Man Lau, Justin W. Tenney

**Affiliations:** School of Pharmacy, Faculty of Medicine, Chinese University of Hong Kong, Shatin, Hong Kong, China; bstc.mandy@hotmail.com

**Keywords:** hospital readmission, drug-disease interaction, potentially inappropriate medications

## Abstract

Early hospital readmission is a common problem among geriatric patients, as they are more susceptible to adverse drug events, which are associated with increased hospital admission. The objective is to examine the association between exposure to potentially inappropriate medications under selected STOPP version 2 criteria related to drug-disease interactions and unplanned early hospitalization within 28 days of index admission in elderly patients prescribed a potentially inappropriate medication. This retrospective single-center study reviewed patients 75 years of age or older that were discharged with 5 or more medications, including at least one selected medication listed in the STOPP version 2 criteria relating to drug-disease interactions. 182 patients, with a mean age of 83.5 years, were included in the study, with anticholinergics being the most common potentially inappropriate medications (22.4%). Potentially inappropriate medications (57.1% vs. 17.1%, *p* < 0.001), gout (31% vs. 11.5%, *p* = 0.003), and gastrointestinal disease (11.9% vs. 2.5%, *p* = 0.026) were shown to increase risk of 28-day readmission, whereas no other factors assessed correlated with readmission. A rapid evaluation of elderly patient discharge medications and concomitant disease states with the aid of the STOPP version 2 criteria could potentially reduce hospital readmissions or emergency department visits.

## 1. Introduction

Unplanned early hospital readmission is a common problem among geriatric patients, and is an indicator of poor patient health outcomes [1,2]. It causes a heavy operational and economic burden on the health care system [1]. Previous studies have found that the proportion of potentially avoidable unplanned readmission varies from 9% to 59%. Of the avoidable readmissions, 45% were drug-related, and the use of potentially inappropriate medications (PIMs) is one of the factors [3,4]. PIM is defined as any medication that poses more risk than benefit, and increases the risk of adverse drug events (ADEs). Other studies have shown the rate of PIMs to be anywhere from 19% to 61% [5,6,7,8,9,10,11]. Due to age-related physiological changes, polypharmacy, and multiple comorbidities, geriatric patients, particularly, are highly susceptible to ADEs. These ADEs are highly associated with hospital readmission, mortality, and elevated healthcare costs [12]. A meta-analysis and systematic review of randomized controlled studies has found evidence that the use of STOPP/START criteria reduces falls, delirium episodes, hospital length-of-stay, care visits, and medication costs [13].

Drug-disease interaction is one of the factors contributing to PIM usage. Surveillance has found that the detected rate of drug-disease interactions ranged from 6% to 30% in elderly patients [14]. Study has shown that prevalence of drug-disease interactions is associated with adverse drug events (ADEs) [15]. It has also been reported that patients prescribed a regimen containing a drug-disease interaction were approximately twice as likely to have self-reported ADEs [16]. However, further research is needed to examine drug-disease interactions’ impact on health outcomes.

Optimization of drug therapy is an important part of elderly care and screening tools have been developed for the detection of PIM(s) in the older population, which includes the widely used Beers criteria and the Screening Tool of Older People’s Prescriptions (STOPP) criteria. Studies analyzing PIMs’ association with adverse drug events (ADEs) and hospitalizations using the Beers criteria have shown conflicting results [16,17]. On the other hand, the STOPP criteria comprise a screening tool developed to assist the identification of PIM(s) and facilitate prevention of ADEs. Studies have shown that the STOPP criteria can effectively detect PIM(s) and help maintain prescription appropriateness [18]. In one prospective study involving 600 elderly patients, results showed that prescription of STOPP PIMs was significantly associated with serious avoidable ADEs, 62.2% of which contributed to hospital admissions [16]. However, whether they can be used to predict adverse outcomes remains unknown. In 2014, STOPP version 2 criteria were published, with expanded criteria based on up-to-date literature review and consensus validation among a European panel of experts; this study focuses on the use of STOPP version 2 criteria [19].

There is a paucity of studies on Asian patients identifying the prevalence of PIM(s) due to drug-disease interactions and PIMs’ effect on health outcomes, such as early hospital readmission. Therefore, a retrospective study was conducted to evaluate the prevalence of PIM(s) caused solely by drug-disease interactions. This was completed by screening patient medical charts with selected STOPP version 2 criteria and exploring the possible association with early hospital readmission in patients over 75 years of age. This is to examine the association between exposure to potentially inappropriate medications under selected STOPP version 2 criteria related to drug-disease interactions and unplanned early hospitalization in the elderly.

## 2. Materials and Methods

A retrospective single-center chart review study was conducted to evaluate the prevalence of PIM(s) caused by drug-disease interactions by screening patient medical records with selected STOPP version 2 criteria and exploring the possible association with early unplanned hospital readmission within 28 days in patients over 75 years of age. Emergency readmission within 28 days was chosen as the study outcome, as it is a commonly used key performance indicator. Eligible patients were identified using Clinical Data Analysis and Reporting System (CDARS). CDARS is an information system with analytical and reporting capacity to support analysis of clinical data, and incorporates the hospital utility (appointments, admission, discharge), diagnosis and drug data of all HA patients. Chart review was conducted utilizing Electronic Patient Records (ePR) by a single reviewer. Patients’ diagnosed diseases, along with any drugs in their active profile that were involved in the development of a drug-disease indication, were documented. The study received local ethics board approval prior to initiation; approval number 2016.689. This study did not require consent from participants, as our study analyzed data retrospectively from usual care during hospitalization. Patients’ records were de-identified prior to analysis.

**Figure 1:**
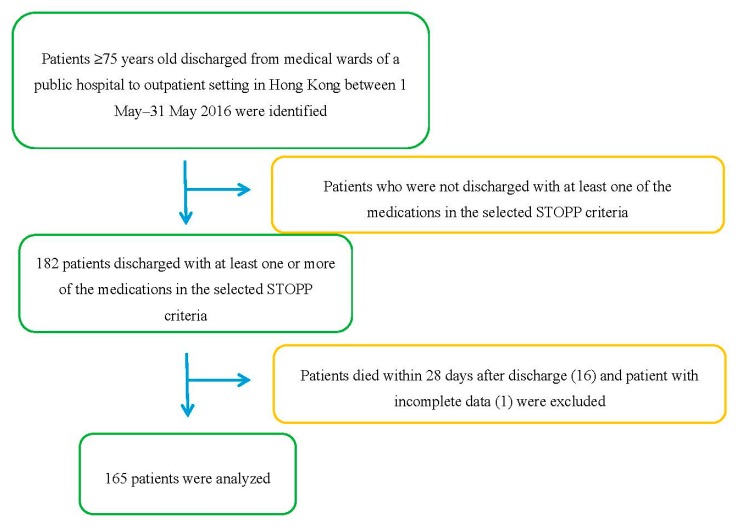
The process of patient selection.

Potentially eligible patients that fulfilled the inclusion criteria and were discharged from medical wards of a public hospital in Hong Kong between 1 May and 31 May 2016 were identified using CDARS. 182 patients were identified. Table 1 lists the inclusion and exclusion criteria for patient recruitment. 16 patients (8.8%) were excluded due to death within 28 days after discharge, and 1 patient was excluded due to incomplete data. A total of 165 patients were analyzed. Figure 1 shows the process of patient selection. For patients who had multiple discharge records within the study period, only the first discharge was taken into account so that data would not be skewed by patients with multiple admissions. Using the formula for the calculation of minimum sample size, a sample size of 107 was sufficient to detect a moderate effect with a significance level of 5% and a power of 80% [20]. Therefore, 165 patients was a sufficient sample size for the detection of statistically significant results in a multiple logistic regression with 3 predictors. Medical records from ePR of the patients were screened and the following data, as shown in Table 2, were obtained.

Statistical analyses were carried out using SPSS package version 24.0. Descriptive statistics are shown as mean ± standard deviation for quantitative data, and frequencies (percentages) for qualitative data. Univariate analyses were carried out using t-test for quantitative variables, and the chi-square test or Fisher’s Exact Test for qualitative variables to compare the patients with and without emergency readmission within 28 days of discharge. A *p* value < 0.05 was considered statistically significant.

Bivariate logistic regression was used to determine the association between categorical variables, including age, gender, number of chronic medication (< or ≥5), number of comorbidities (< or ≥5), presence of PIM(s) as identified by STOPP criteria, the type of PIM(s) identified by STOPP criteria, and early readmission. A *p* value < 0.05 was considered statistically significant. Variables with *p* value < 0.05 were selected to be included in the multiple logistic regression model.

Multiple logistic regression was carried out to determine the correlation between the presence or the absence of PIM(s) related to drug-disease interactions identified by selected STOPP criteria and early emergency readmission. The adjusted odds ratio was obtained in the multiple regression model that included gastrointestinal disorder and gout as the cofounding factors. Variables with *p* value < 0.05 were considered statistically significant.

## 3. Results

Overall, 182 patients with a mean age of 83.5 years were screened. There was no significant difference in age, gender, number of chronic medications and number of comorbidities between patients that had unplanned readmission and the remainder of the analysis, as shown in Table A1. Of the 17 patients that were excluded, 1 was due to incomplete data and 16 were due to death within 28 days of discharge. Of the 165 patients included in the analysis, 42 patients (25.5%) had unplanned readmission within 28 days of discharge. The proportion of patients with unplanned readmission within 28 days had significantly more PIM(s) identified by STOPP version 2 criteria (57.1% vs. 17.1%, *p* < 0.001), more PIM(s) related with the use of anticholinergics (52.4% vs. 12.2%, *p* < 0.001), more prescription of oral bisphosphonate in patients with history of gastrointestinal disease (7.1% vs. 0%, *p* = 0.016), and more patients with gastrointestinal disorder (11.9% vs. 2.5%, *p* = 0.026) and gout (31% vs. 11.5%, *p* = 0.003). Drug-disease interactions with anticholinergics were the mostly identified PIMs, with a prevalence of 27.3% among the whole study population and 57.1% among patients with early readmissions. Table A1 shows a further breakdown of the different drug-disease interactions in patients who were and were not readmitted within 28 days.

### 3.1. Variables Associated with Unplanned Early Readmission

#### 3.1.1. Bivariate Analysis

Unplanned readmissions within 28 days were significantly more frequent in patients with PIM(s) identified by STOPP version 2 criteria (OR 6.476; 95% CI 2.996, 13.988; *p* < 0.001), PIM(s) related to the use of anticholinergics (OR 12.667; 95% CI 5.103, 31.438; *p* < 0.001), gastrointestinal disorder (OR 5.405, 95% CI 1.233, 23.699; *p* = 0.025), and gout (OR 3.490; 95% CI 1.479, 8.238; *p* = 0.004).

No significant differences were found between age (OR 1.447; 95% CI 0.711, 2.944; *p* = 0.308), gender (OR 0.723; 95% CI 0.356, 1.469; *p* = 0.370), number of chronic medications (OR 1.517; 95% CI 0.660, 3.490; *p* = 0.327), or number of comorbidities (OR 0.993; 95% CI 0.486, 2.026; *p* = 0.984). Table 4 shows the results of a simple logistic regression analysis of the association between early hospital readmission within 28 days and various factors.

#### 3.1.2. Multivariate Analysis

Multiple logistic regression was performed with identification of PIM(s) by STOPP version 2 criteria, gastrointestinal disorder and gout as independent variables, and early hospital readmission as the dependent variable to analyze the association between the identification of PIM(s) and the three independent variables, as shown in Table 5. PIM(s) related to the use of anticholinergic medications was not included in the multiple logistic regression model, as it directly contributes to the identification of PIM(s) by STOPP version 2 criteria. Early unplanned readmission within 28 days was significantly related with identification of PIM(s) by STOPP criteria (OR 6.557; 95% CI 2.889, 14.971; *p* < 0.001) and gout (OR 4.344; 95% CI 1.666, 11.323; *p* = 0.003). The Hosmer-Lemeshow Goodness-of-Fit Test indicated that the model was well calibrated (*p* > 0.05).

## 4. Discussion

As the STOPP version 2 criteria are not organized by drug-disease state interaction, no high-quality study has specifically looked at drug-disease interactions listed in the STOPP criteria separately. Use of the Beers Criteria in European nursing home residents has shown an incidence rate of drug-disease interactions of 19–55% [9,21,22,23,24]. This study provides supporting evidence that, although it may not be possible to perform a comprehensive STOPP criteria evaluation, even a partial evaluation can be potentially beneficial in reducing hospital readmissions.

In screening patients for inclusion in the study, we identified an overall PIM(s) rate of 27.3%, which is fairly similar to rates reported in other literature, although the other studies looked at all STOPP criteria, instead of just focusing on drug-disease interactions, and did not require one of the PIM(s) related to drug-disease interactions to be included on all patients enrolled [10,12]. Also, due to the limitations of the drug formulary at the study site, not all the medications listed on the selected STOPP version 2 criteria were involved in the development of drug interactions.

It is unknown if bringing these drug-disease interactions to the attention of the prescriber will result in the prescriber discontinuing or changing the drug therapy. A study by Gill et al. [25] reported that 37.9% of PIM(s) identified in the long-term care setting were discontinued by the prescriber when brought to their attention. In a population of ambulatory elderly patients with cancer, a pharmacist-led medication review was shown to facilitate identification of PP and PIMs [26]. Previous literature, much like our study, has shown anticholinergic drug-disease interactions to be the most common drug-disease PIM identified. Our study supports previous literature that shows the prescribing antimuscarinics in elderly frequently results in drug-disease interactions [27].

Combining screening systems has shown higher PIM identification rates, but the more screening systems implemented, the more time-consuming the screening process will be, and this may not be practical in common practice [11]. Our study accounts for the limitation that a significant portion of hospitals have of issues with understaffing [28]. Evaluating all 114 STOPP criteria can be time consuming and challenging, with adequate staffing and resources being the significant barriers. Moreover, additional information beyond the medication list is needed for 85% of the STOPP criteria. Therefore, only selected STOPP criteria related to drug-disease interaction were applied in this study, to account for the limited resources in the majority of the public hospitals in Hong Kong. This study involved the screening of 165 patients, and the screening process was completed within 30 h by a single pharmacist, which showed an efficient patient screening process. The STOPP criteria have been shown to be more effective at identifying PIM(s) than Beers criteria. However, the STOPP criteria often require medical history more than Beers criteria (67% vs. 31%).

There are several limitations to our study. Firstly, our study is retrospective in nature, and the potential impact of a concurrent review of patient medications cannot be assessed based solely on this one study with a retrospective design, further prospective studies are needed to confirm results and potential impact. Secondly, our study is a single-center study, which limits its generalizability to other hospitals and regions. Next, as medications listed in the STOPP criteria vary in availability based on the country, not all medications listed in the drug-disease specific STOPP version 2 criteria could be assessed, as they are not all commercially available in Hong Kong. The fourth limitation of the study is the small sample size, although this did not prevent our study from showing statistically significant results regarding PIM(s), gout, or gastrointestinal disease and 28-day readmission. Another limitation is the lack of real assessment of the clinical signs of patients; for example, we were unable to gauge the severity of certain disease states, such as level of severity of urinary retention or intraocular pressure in open-angle glaucoma.

It was shown that avoiding PIM(s) reduces drug-related morbidity and mortality in elderly patients [29]. The use of explicit screening tools, including the simplest and easiest method of detecting PIM(s), is expected to improve the quality of prescription [30]. Comprehensive evaluation with any of these tools is quite time consuming, and can be difficult to integrate into a busy workflow in the healthcare setting. Clinical decision support systems are one alternative, but can be difficult to design and implement due to limitations in terms of the lack of specificity of the criteria [31]. A more simplistic system may not be as comprehensive or provide as much benefit, but could still improve patient outcomes compared to no review at all. Successful narrow-scope services may later be expanded into more comprehensive clinical services. With current technological advancements, the development of software for drug-use screening and intervention may be possible. Currently, SENATOR, a European Union-funded project, is developing a new software engine for the assessment and optimization of drug therapy in the older population based largely on STOPP criteria [32]. SENATOR software is undergoing international randomized controlled trial to test its efficacy in optimizing drug use and reducing ADE incidence.

## 5. Conclusions

The prevalence of PIM(s) use is high in the elderly population, and it was found that 27.3% of the elderly population in a public hospital in Hong Kong were dispensed at least one or more PIM(s). The use of PIM(s) in the elderly is associated with adverse clinical outcomes, such as increased ADEs, mortality, morbidity, and increased healthcare resource utilization [33]. This study shows that the use of PIM(s) is associated with early hospital readmission, which is an adverse clinical outcome and contributes to heavy burden to our healthcare system. There is a need for interventions to improve drug use in elderly patients, which can be aided by the use of STOPP criteria.

There are still inadequate studies on the application of STOPP criteria as an intervention tool for improving medication appropriateness, and further evidence is needed to evaluate the clinical benefit of their application and effectiveness. In addition, due to limited healthcare resources and manpower, there is difficulty in the application of comprehensive patient screening and intervention with STOPP criteria.

## Figures and Tables

**Table 1:** Inclusion and exclusion criteria.

**Inclusion Criteria**	
➢ ≥75 years old	
➢ Patient discharged with one or more of the following medications:	
➢ Verapamil/Diltiazem ➢ Tricyclic Antidepressants ➢ Chlorpromazine/Clozapine/flupenthixol/zuclopenthixol * ➢ Antipsychotics except quetiapine or clozapine ➢ Anticholinergics/antimuscarinics ➢ Prochlorperazine/Metoclopramide	➢ Anti-muscarinic bronchodilators ➢ Non-selective beta-blocker ➢ COX-2 selective NSAID ➢ Oral bisphosphonate ➢ Thiazolidinedione ➢ Oestrogens
➢ Discharged to outpatient setting	
**Exclusion Criteria**	
➢ <5 chronic medications	
➢ Death within 28 days after discharge	

* Fluphenzine, pipothiazine, promazine are also included in the STOPP criteria, but are not commercially available in Hong Kong so were not incorporated into the inclusion criteria.

**Table 2:** Data collected from medical record screening.

Type of Data	Data Collected
Demographic data	Age, gender
Medications	Number of chronic medications, identified PIM(s) related to drug-disease interactions under selected STOPP version 2 criteria as shown in Table 3
Comorbidities	Type of comorbidities, number of comorbidities
Readmission	Records of emergency readmission within 28 days of discharge

**Table 3:** PIMs related to drug-disease interactions under selected STOPP version 2 criteria.

Drugs	Interacting Disease State
Diltiazem or verapamil	NYHA class III or IV heart failure
Tricyclic antidepressants (TCAs)	dementia, narrow angle glaucoma, cardiac conduction abnormalities, prostatism, prior history of urinary retention
Chlorpromazine, Clozapine, flupenthixol, zuclopenthixol *	History of prostatism or previous urinary retention
Antipsychotics (i.e., other than quetiapine or clozapine)	Parkinsonism, Lewy Body disease
Anticholinergics/antimuscarinics	Dementia, delirium, chronic cognitive impairment narrow angle glaucoma, chronic prostatism
Prochlorperazine or metoclopramide	Parkinsonism
Antimuscarinic bronchodilators	History of narrow angle glaucoma or bladder outflow obstruction
Non-selective beta blocker	History of asthma requiring treatment
COX-2 NSAID (celecoxib, etoricoxib, parecoxib)	Cardiovascular disease
Oral bisphosphonate	Gastrointestinal disease
Oestrogens	History of breast cancer or venous thromboembolism (VTE)
Thiazolidenediones (Pioglitazone, Rosiglitazone)	Heart Failure

* Fluphenzine, pipothiazine, promazine are also included in the STOPP criteria, but are not commercially available in Hong Kong so were not incorporated into the inclusion criteria.

**Table 4:** Simple logistic regression analysis of the association between early hospital readmission within 28 days and various factors.

Possible Factors Influencing Readmission		Raw OR (95% CI)	*p*
Age	<83 years old	1.447 (0.711, 2.944)	0.308
	≥83 years old		
Gender	Male	0.723 (0.356, 1.469)	0.370
	Female		
Number of chronic medications	<6	1.517 (0.660, 3.490)	0.327
	≥6		
Number of comorbidities	<5	0.993 (0.486, 2.026)	0.984
	≥5		
PIM(s) identified	Yes	6.476 (2.996, 13.988)	<0.001
	No		
PIM(s) associated with the use of			
TCAs		1.476 (0.130, 16.702)	0.753
Anticholinergics/antimuscarinics		7.920 (3.518, 17.828)	<0.001
Antimuscarinic bronchodilators		2.484 (0.635, 9.724)	0.191
Arrhythmia		0.507 (0.215, 1.196)	0.121
Asthma		2 (0.323, 12.401)	0.457
Cancer		1.298 (0.464, 3.627)	0.619
COPD		0.758 (0.237, 2.425)	0.640
Delirium		1.072 (0.322, 3.566)	0.910
Dementia		0.576 (0.065, 5.073)	0.619
Diabetes		0.713 (0.348, 1.459)	0.354
Gastrointestinal disorder		5.405 (1.233, 23.699)	0.025
Glaucoma		2.053 (0.550, 7.661)	0.285
Gout		3.490 (1.479, 8.238)	0.004
Heart failure		1.492 (0.672, 3.312)	0.325
Hyperlipidemia		0.809 (0.387, 1.690)	0.572
Hypertension		0.805 (0.385, 1.683)	0.564
Ischemic heart disease		0.777 (0.367, 1.645)	0.510
Liver disease		0.712 (0.191, 2.655)	0.612
Myocardial infarction		1.527 (0.492, 4.755)	0.465
Parkinsonism		0.576 (0.065, 5.073)	0.619
Prostatism		0.576 (0.184, 1.802)	0.343
Psychiatric disorder		0.713 (0.348, 1.459)	0.354
Renal disease		1.836 (0.815, 4.140)	0.143
Stroke		1.095 (0.424, 2.823)	0.851
Urine retention		1.338 (0.509, 3.517)	0.555

**Table 5:** Multiple logistic regression analysis of the association between early hospital readmission within 28 days and three factors (PIM(s) identified, gastrointestinal disorder and gout).

Readmission Factors	Adjusted OR (95%CI)	*p* Value
PIM(s) identified	6.557 (2.889, 14.971)	<0.000
Gastrointestinal disorder	3.718 (0.742, 18.622)	0.110
Gout	4.344 (1.666, 11.323)	0.003

## Appendix A

**Table A1:** Characteristics of the study population.

Characteristic		Total (*n* = 165) (%)	Not Readmitted within 28 Days (*n* = 123) (%)	Readmitted within 28 Days (*n* = 42) (%)	*p* Value
Age		83.35 ± 5.49	83.03 ± 5.33	84.29 ± 5.84	0.383
Gender	Male	65 (39.4)	46 (37.4)	19 (45.2)	0.369
	Female	100 (60.6)	77 (62.6)	23 (54.8)	
Number of chronic medication	<6	45 (27.3)	36 (29.5)	9 (21.4)	0.575
	6–10	104 (63)	76 (62.3)	28 (6.7)	
	>10	16 (9.7)	11 (9)	5 (11.9)	
Number of comorbidities	<5	98 (59.4)	73 (59.8)	25 (59.5)	0.984
	≥5	67 (40.6)	50 (41)	17 (40.5)	
Patients with PIM(s) identified		45 (27.3)	21 (17.1)	24 (57.1)	<0.001
Arrhythmia		47 (28.5)	39 (32)	8 (19)	0.117
Asthma		5 (3)	3 (2.5)	2 (4.8)	0.602
Cancer		20 (12.1)	14 (11.5)	6 (14.3)	0.619
COPD		19 (11.5)	15 (12.3)	4 (9.5)	0.784
Delirium		15 (9.1)	11 (9)	4 (9.5)	1.000
Dementia		6 (3.6)	5 (4)	1 (2.4)	1.000
Diabetes		73 (44.2)	57 (46.7)	16 (38.1)	0.353
Gastrointestinal disorder		8 (4.8)	3 (2.5)	5 (11.9)	0.026
Glaucoma		10 (6.1)	6 (4.9)	4 (9.5)	0.278
Gout		27 (16.4)	14 (11.5)	13 (31)	0.003
Heart failure		38 (23)	26 (21.3)	12 (28.6)	0.323
Hyperlipidemia		61 (37)	47 (38.5)	14 (33.3)	0.572
Hypertension		112 (67.9)	85 (69.7)	27 (64.3)	0.564
Ischemic heart disease		58 (35.2)	45 (36.9)	13 (31)	0.509
Liver disease		15 (9.1)	12 (9.8)	3 (7.1)	0.762
Myocardial infarction		15 (9.1)	10 (8.2)	5 (11.9)	0.535
Parkinsonism		6 (3.6)	5 (4.1)	1 (2.4)	1.000
Prostatism		23 (13.9)	19 (15.6)	4 (9.5)	0.339
Psychiatric disorder		73 (44.2)	57 (46.7)	16 (38.1)	0.353
Renal disease		34 (20.6)	22 (18)	12 (28.6)	0.139
Stroke		26 (15.8)	19 (15.6)	7 (16.7)	0.851
Urine retention		23 (13.9)	16 (13.1)	7 (16.7)	0.554
Drug-disease interaction with					
Selective COX-2 NSAIDs/Diltiazem/VerapamilNon-selective beta blockers/Oestrogens/Thiazolidinediones		0 (0)	0 (0)	0 (0)	-
TCAs		3 (1.8)	2 (1.6)	1 (2.4)	1
Antipsychotics		1 (0.6)	1 (0.8)	0 (0)	1
Anticholinergics/antimuscarinics		37 (22.4)	15 (12.2)	22 (52.4)	<0.001
Antimuscarinic bronchodilators		9 (5.5)	5 (4.1)	4 (9.5)	0.234
Prochlorperazine/Metoclopramide		1 (0.6)	0 (0)	1 (2.4)	0.255
Oral bisphosphonate		3 (1.8)	0 (0)	3 (7.1)	0.016
